# Stool metatranscriptomics: A technical guideline for mRNA stabilisation and isolation

**DOI:** 10.1186/s12864-015-1694-y

**Published:** 2015-07-04

**Authors:** Michael Reck, Jürgen Tomasch, Zhiluo Deng, Michael Jarek, Peter Husemann, Irene Wagner-Döbler, On behalf of COMBACTE consortium

**Affiliations:** Research Group Microbial Communication, Helmholtz Centre for Infection Research, Braunschweig, Germany; Research Group Genome Analysis, Helmholtz Centre for Infection Research, Braunschweig, Germany

**Keywords:** Metatranscriptomics, Gut microbiota, RNA stabilisation, RNA Later, RNA Protect, All Protect metagenomics, Stool

## Abstract

**Background:**

The complex microbiome of the gut has an enormous impact on human health. Analysis of the transcriptional activity of microorganisms through mRNA sequencing (metatranscriptomics) opens a completely new window into their activity *in vivo,* but it is highly challenging due to numerous technical and bioinformatical obstacles. Here we present an optimized pipeline for extraction of high quality mRNA from stool samples.

**Results:**

Comparison of three commercially available RNA extraction kits with the method of Zoetendal revealed that the Powermicrobiome Kit (MoBio) performed best with respect to RNA yield and purity. Next, the influence of the stabilization reagent during sample storage for up to 15 days was studied. RIN analysis and qRT-PCR of spiked-in and indigenous genes revealed that RNA Later preserved mRNA integrity most efficiently, while samples conserved in RNA Protect showed substantial mRNA decay. Using the optimized pipeline developed here, recovery rates for spiked-in *E.coli* cells expressing fluorescing proteins were 8.7-9.7 % for SuperfolderGFP and 14.7-17.8 % for mCherry. The mRNA of stabilized stool samples as well as of snap-frozen controls was sequenced with Illumina Hiseq, yielding on average 74 million reads per sample. PCoA analysis, taxonomic classification using Kraken and functional classification using bwa showed that the transcriptomes of samples conserved in RNA Later were unchanged for up to 6 days even at room temperature, while RNA Protect was inefficient for storage durations exceeding 24 h. However, our data indicate that RNA Later introduces a bias which is then maintained throughout storage, while RNA Protect conserved samples are initially more similar to the snap frozen controls. RNA Later conserved samples had a reduced abundance of e.g. Prevotellaceae transcripts and were depleted for e.g. COG category “Carbohydrate transport and metabolism”.

**Conclusion:**

Since the overall similarity between all stool transcriptional profiles studied here was >0.92, these differences are unlikely to affect global comparisons, but should be taken into account when rare but critically important members of the stool microbiome are being studied.

**Electronic supplementary material:**

The online version of this article (doi:10.1186/s12864-015-1694-y) contains supplementary material, which is available to authorized users.

## Background

The influence of the microbiota inhabiting the human body on an individual’s health has become a major research topic and enormous efforts are made to address this question [1]. However, shifts in the composition of the microbial community provide little or only very global information about the metabolic activities of the microbes, which are key for understanding their actual roles in health and disease. Metatranscriptomics, in particular when combined with other meta-omics approaches, is capable of addressing this. So far metatranscriptome studies are technically and bioinformatically highly challenging and thus the technique is still in its infancy.

In a first metatranscriptome study Turnbaugh *et al.* evaluated the transcriptional diversity of the gut microbiomes of a monozygotic twin pair [2]. Maurice *at al.* [3] and Perez-Cobas *et al.* [4] provided a proof of concept with their metatranscriptome studies on the disturbance of the gut microbiota during treatment with xenobiotics (antibiotics). In a multi-omics approach Perez-Cobas *et al.* analysed changes of the total and active metagenome (16S rDNA and rRNA), metabolome, metatranscriptome and metaproteome during treatment of one patient with a common ß-lactam antibiotic. Franzosa *et al.* [5] used stool samples originating from 8 healthy donors of a prospective cohort study to evaluate the relationship between the metagenomes and the metatranscriptomes. Interestingly this study showed that across subjects, metatranscriptomic functional profiles were more individualized than the corresponding metagenomic profiles or 16S rRNA gene diversity. Despite the potential of metatranscriptomic analysis of the GIT microbiota, the technical challenges are numerous and even the best bioinformatic approaches cannot overcome poor biological sample quality and processing artefacts. The short half-lives of mRNA [6], a high content of nucleases present in stool samples [7], ineffective cell lysis [8], high amounts of inhibitory substances co-extracted with the RNA [7;8] and difficult enrichment of bacterial mRNA [9, 10] can be huge hurdles on the way to a successful metatranscriptome study. First metatranscriptome studies of the human gut suffered e.g. from ineffective mRNA enrichment and thus a potential loss of low abundant transcripts due to low sequencing depth [2, 11].

Thus, in this study we focused on the technical optimization of RNA stabilization and extraction to provide high quality RNA for high throughput RNA sequencing for stool metatranscriptome analysis. We compared 3 different commercially available RNA extraction kits with the method of Zoetendal *et al.* [12] with respect to RNA yield and RNA quality. Since application of liquid nitrogen is often not feasible in clinical practice, the choice of an appropriate stabilizing agent is crucial to prevent RNA degradation. Therefore, we studied the effect of the stabilization reagent (RNA protect or RNA Later) and storage temperature on the decay of mRNA and rRNA for 15 days using quantitative RT-PCR of indigenous stool genes as well as of spike-in controls. Control samples immediately frozen in liquid nitrogen provided the gold standard. Using *E. coli* spike-in cells overexpressing mCherry and sFGFP we demonstrate a high absolute recovery rate of mRNA when applying our optimized protocol.

Samples were then subject to full strand specific mRNA sequencing on an Illumina HiSeq2000 platform to detect shifts in the community transcriptome caused by the stabilizing reagent, storage time or storage temperature. Sequencing reads were functionally and taxonomically assigned using the Burrows-Wheeler alignment tool (bwa) [13] against the Human Microbiome Project database. Additionally we utilized the new Kraken software to assign taxonomic labels to the sequencing reads and compared the results with the taxonomic classification performed with bwa. The metatranscriptomes were analyzed for shifts in the taxonomic and functional profiles and compared to control samples immediately frozen in liquid nitrogen.

Our data provide high resolution information on the stability of the metatranscriptome in stool samples under various preservation and storage conditions. The analyses were conducted on sub-samples from one stool sample from a healthy donor to exclude the variability introduced by differences in stool composition from different donors. Twelve deeply sequenced metatranscriptomes were obtained, reflecting four different storage conditions and 3 time-points. The data can be used to plan proper sampling, conservation and processing of stool samples for metatranscriptomics. This might be particularly relevant for clinical trials and cohort studies where experimental demands need to be compatible with high sample throughput and routine clinical practice.

## Results and discussion

### Comparison of different RNA extraction procedures for stool samples

For optimizing RNA extraction from stool samples, we tested different commercially available kits (Stool Total RNA Purification Kit (Norgen), Powermicrobiome RNA Isolation Kit (MoBio) and RNeasy Mini Kit (Qiagen)) and compared them with the method established by Zoetendal *et al.* [12]. The latter protocol is based on a classical phenol-chloroform extraction procedure and represents the gold standard for isolating RNA from stool.

The same sample pretreatment was used in all cases (see Additional file 1: Figure S1), and all samples were conserved in RNA Later. Mechanical lysis was accomplished using the Fast Prep instrument and zirconia beads (0.1 mm diameter). We utilized a phenol/chloroform/isoamylalcohol mixture to stabilize the RNA during bead beating. The following modifications were made: For the RNeasy Kit (Qiagen) we used a combination of enzymatic lysis (LM solution) and mechanical cell disruption by vortexing with zirconia beads (0.1 mm) instead of bead-beating. This procedure is routinely used in our lab to lyse streptococci. The Norgen Kit was used either with the glass beads supplied by the manufacturer (unmodified procedure) or with 0.1 mm zirconia beads (modified procedure), which are also utilized in the Zoetendal protocol. Due to their smaller size (0.1 mm) they are superior for the rupture of bacterial cells.

**Fig. 1:**
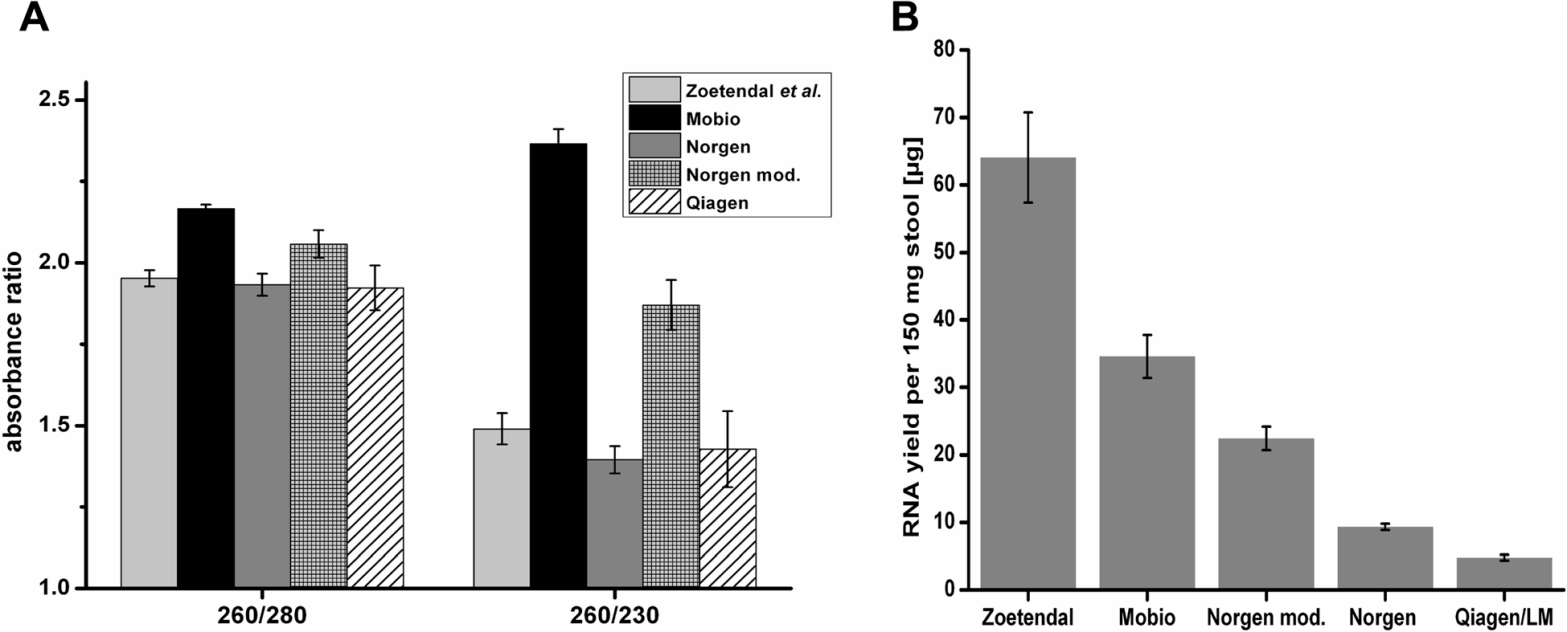
Quality and yield of total RNA extracted from stool samples using four different commercially available kits in comparison to the Zoetendal method. **a** Absorbance ratios 260/280 and 260/230; (**b**) Total RNA yield from 150 mg stool (wet weight)

In Fig. 1a the purity of the isolated RNA is shown. Low values for the absorbance ratio 260/280 are indicative of protein contamination. All extracted RNAs showed ratios close to the optimal value of 2. Low values for the 260/230 ratio are indicative of contamination with salts, organic solvents and other inhibitory substances present in stool samples (e. g. bile salts, humic acids). RNAs isolated with the MoBio Kit had significantly higher 260/230 ratios than RNAs extracted with the other protocols. Thus based on the absorption ratios the quality of the RNAs extracted with the MoBio Kit was superior to all other RNA extraction procedures tested.

The RNA yield per 150 mg of stool sample (wet weight) for the different extraction protocols is shown in Fig. 1b. With the Zoetendal protocol, more than 60 μg of total RNA were obtained, providing the highest RNA yield of all tested procedures. The RNA yield of the MoBio Kit (approx. 35 μg) was higher than that of the Norgen Kit (approx. 10 μg with the original and 25 μg with the modified procedure, respectively). The lowest yield (approx. 7 μg) was obtained using the Qiagen RNeasy Kit and our routine RNA extraction protocol for Streptococci. Although the Zoetendal protocol resulted in the highest RNA yield, the MoBio protocol was faster, less sensitive to handling errors and thus more reproducible, and the resulting RNA had a better quality. Thus, the MoBio Kit performed best and was implemented in our pipeline for stool metatranscriptomics.

Highly effective extraction methods are necessary to retrieve the entire metatranscriptome of a sample. The efficiency of cell lysis may vary considerably among different species of bacteria. For example, the cell wall of Firmicutes, one of the two dominant phyla colonizing the human gut, consists of multiple layers of peptidoglycan and is therefore hard to lyse. Lakay *et al.* [8] compared different cell lysis methods and showed that bead-beating is more efficient than methods involving enzymatic lysis, liquid nitrogen grinding or microwave based rupture. The lower yield of our laboratory protocol routinely applied for the lysis of streptococci is therefore caused by the inefficiency of vortexing compared to bead-beating. In the finally chosen protocol for stool metatranscriptomics (MoBio Kit) a bead-beating step is combined with chemical lysis.

**Fig. 2:**
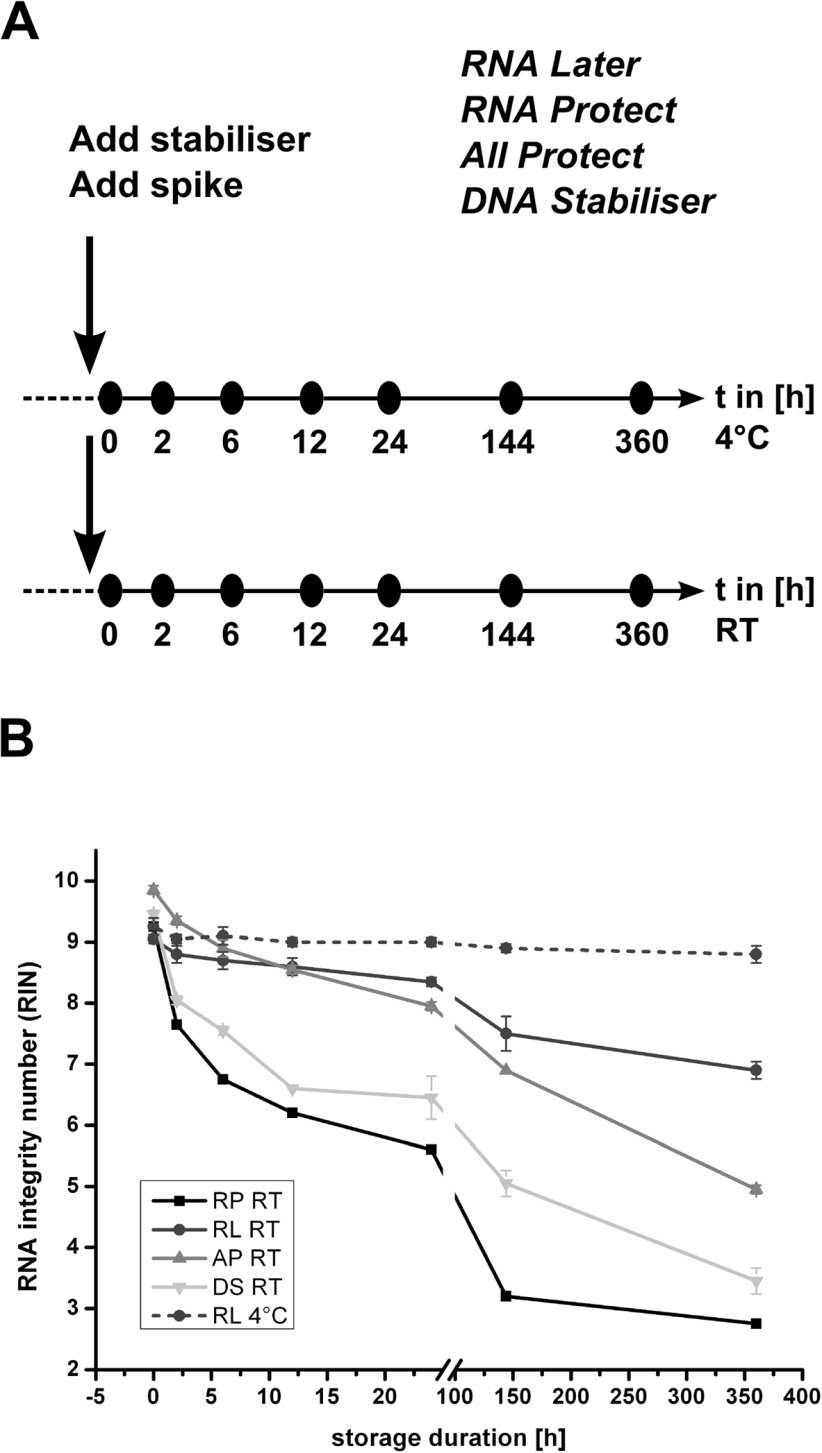
Effect of stabilization reagent, storage time and storage temperature on RNA integrity. Experimental design (**a**) and RNA integrity (**b**) of total RNA extracted from stool samples conserved in four different stabilizing reagents for up to 15 days at room temperature and 4 °C

Stool samples contain high amounts of inhibitory substances like humic acids, bile salts, billirubins and complex carbohydrates [14, 15] which interfere with downstream applications like quantitative PCR, mRNA enrichment, cDNA synthesis or RNA labeling for microarray analysis [16]. In particular the mRNA enrichment step using subtractive hybridization methods is salt sensitive [16]. Efficient removal of these substances is thus crucial for a successful RNA extraction protocol applicable for metatranscriptomics. RNA extracted using the MoBio Kit showed the highest purity. The RNAs extracted with our protocol showed higher RIN values (all above 9 at t = 0, see Fig. 2b) than RNAs from comparable studies [5, 7], indicating effective RNA conservation during the complete extraction procedure.

### Comparison of different stabilizing reagents for stool samples

The stability of RNA is a critical factor for metatranscriptome analyses since both in the clinic and in private settings (e.g., self-sampling during cohort studies) stool samples can often not be transferred to −70 °C or −20 °C freezers immediately. Thus the transfer time between conservation of the stool sample in a stabilizing reagent and its arrival at −70 °C is a crucial and variable factor. An ideal stabilizing reagent should prevent RNA degradation even at room temperature for at least several hours. We therefore compared RNA stability in 3 different RNA stabilizing reagents (Stool Total RNA Purification Kit (Norgen), Powermicrobiome RNA Isolation Kit (MoBio) and RNeasy Mini Kit (Qiagen)) at two different storage temperatures (4 °C and RT) for up to 360 h. In addition, the DNA stabilizer solution of the PSP Spin Stool DNA Plus Kit (Stratec, Germany) was also included since this kit would allow to simultaneously isolate RNA and DNA. Allprotect allows the simultaneous analysis of the metabolom, transcriptome and proteome and is therefore of particular interest for multi-omics approaches. RNA Later is also used in the Zoetendal procedure [12]. The experimental design is shown in Fig. 2a. The original stool sample was suspended in the respective stabilizing reagent, spike-in controls were added and the samples maintained at the indicated temperature until analysis up to 15 days (360 h) later. As an external control to monitor and quantify the RNA decay, we spiked the samples with IPTG-induced *E. coli* cells expressing mCherry and GFP under the control of the Lac promoter. The amount of the two spikes was calculated to represent approximately 0.9 % each of the total cell number in the sample, assuming that 1 g stool contains approximately 10^11^ cells [17]. Control samples derived from the same faecium were immediately snap-frozen in liquid nitrogen. These samples contained no spike-in controls and represent the initial transcriptional profile. RNA from all samples was extracted using the MoBio Kit as described above.

### RNA integrity of total stool RNAs

The integrity of the isolated RNA was determined using the Bionalyzer (Agilent). RNA integrity numbers (RINs) for the different RNA extraction methods are shown in Fig. 2b. All RNAs extracted immediately after resuspension of the sample in the stabilizer (0 h) showed comparable high RIN numbers above 9, which indicates intact RNA of high quality. RNA decay during storage showed large differences depending on the storage temperature and stabilizing reagent. The steepest decrease in RIN number was observed for the samples conserved in RNA Protect at room temperature. After 6 days the RIN decreased from 9.3 to values around 3. RNA extracted from stool samples conserved in the DNA stabilizer showed a similar behaviour but was slightly more stable. RNA Later and Allprotect prevented RNA degradation much more efficiently. RNA Later performed best: Even after 15 days of storage at room temperature the extracted RNAs showed RIN numbers above 7. To demonstrate the influence of the storage temperature on RNA quality, we also determined the RINs of RNA extracted from samples stored at 4 °C in RNA Later. Storage at 4 °C in RNA Later almost completely prevented RNA degradation. No significant decrease in RIN was observed even after 15 days of storage.

### Stability of mRNA determined by quantitative RT-PCR of spike-ins and indigenous highly expressed genes

The RIN numbers determined by the Bioanalyzer largely reflect rRNA integrity and thus do not necessarily correspond to the integrity of mRNAs, although it is usually assumed that both are highly correlated. To quantify the mRNA decay we used quantitative reverse transcription PCR (qRT-PCR). Primers targeting the two spike-in controls, mCherry and sFGFP, were designed. In addition the copy number of the glyceraldehyde 3-phosphate dehydrogenase (GAPDH) mRNA of *Faecalibacterium prausnitzii* was determined. To quantify the degradation of ribosomal RNA a primer pair targeting the 23S rRNA was used (see Additional file 2: Table S1).

**Fig. 3:**
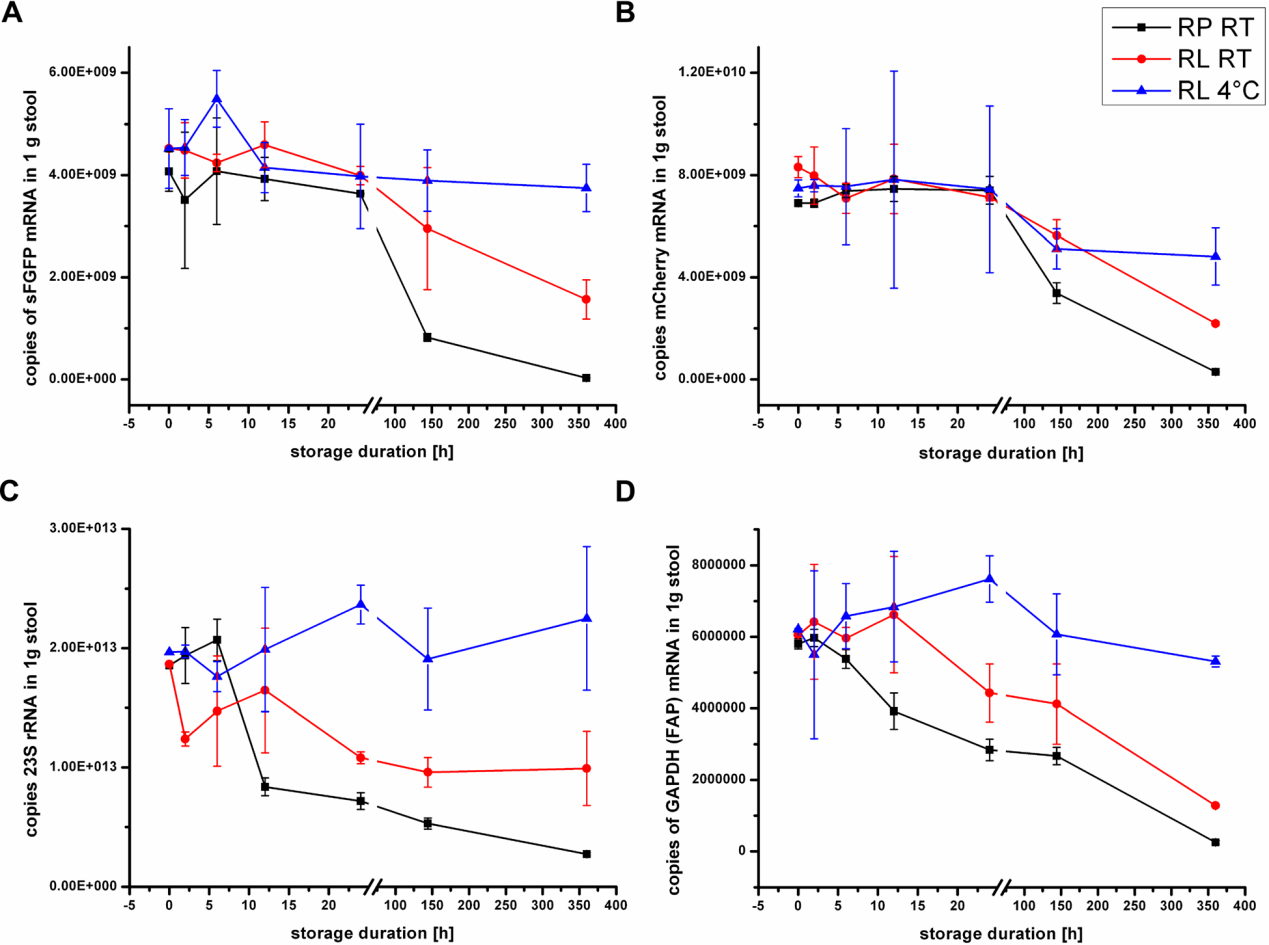
Stability of transcripts from spike-ins and indigenous targets in stool mRNA for up to 15 days. Absolute copy numbers of the spikes sFGFP (**a**) and mCherry (**b**) and of the two indigenous targets 23S rRNA gene (**c**) and GAPDH gene (**d**) as determined by quantitative RT-PCR in stool samples. Samples were stored in RNA Later at room temperature (red graph) or at 4 °C (blue graph) or in RNA Protect at RT (black graph)

Even though the RNAs derived from samples stored in RNA Protect for 144 h were highly degraded (RIN 3.3), we initially found no significant decrease in RNA copy number for any of our chosen targets (data not shown). The primers used for qRT-PCR amplified approximately 100 bp of the target gene. The electropherograms from the Bioanalyzer showed a smear of degraded RNA in a size range of 100–200 bp (data not shown). Thus this degraded RNA may still be reverse transcribed and consequently function as a PCR target. We therefore tested how the length of the amplified mRNA affected its recovery in the qRT-PCR analysis. In Additional file 3: Figure S2 the detected copy numbers for the sFGFP-spike for samples stabilized in RNA Protect and extracted at t = 0 and after 144 h of storage at RT are shown for primers amplifying approx. 100, 300, 500 and 700 bp of the sFGFP gene, respectively. The differences in transcript copy numbers between t = 0 h and t = 144 h were more pronounced for primer pairs amplifying a longer region of the target. Accordingly utilization of primers which amplify a longer portion of the target gene is a more sensitive measure for mRNA decay. Utilization of primers amplifying more than 500 bp of the target gene did not increase the sensitivity of the assay any further. Consequently we used primers amplifying approximately 500 bp of the target gene for further analysis. Notably, amplification efficiency was not significantly influenced (data not shown). Using these new primers the expected significant decrease in the copy number for samples stored in RNA Protect for a longer period was found (Fig. 3).

The overall trend of RNA decay among the different storage conditions was the same for all targets and confirmed the RIN analysis. Storage at 4 °C in RNA Later conserved the RNA almost completely. The copy number of all RNA targets analyzed was stable even after 15 days of storage. Even at RT, the RNA of stool samples stored in RNA Later was stable for 24 h to 144 h. In contrast, RNA stored in RNA Protect degraded rapidly. The copy numbers for the 4 targets decreased strongly after 144 h. At RT, the copy numbers of the indigenous targets GAPH and the 23S rRNA were significantly reduced already after 24 h, while both spike-ins were unchanged, possibly because of their higher total abundance. The data are in accordance with the RNA integrity analysis using the Bioanalyzer and confirm a good correlation between the stability of rRNA and mRNA. Among the tested stabilizing reagents RNA Later thus is the reagent of choice to conserve stool sample RNA at ambient temperature.

### Extraction efficiency of mRNA determined by calculation of spike-in recovery

To estimate the recovery rate of the spike-in controls we compared the theoretical number of mRNA copies spiked into the stool samples within *E. coli* cells with the absolute number of copies determined by qRT-PCR. Assuming that 1 OD_600_ corresponds to 8*10^8^*E. coli* cells/ml culture in rich media [18], approximately 0.9*10^9^*E. coli* cells were spiked per gram stool into each sample. This corresponds to approx. 0.9 % of the total cell number (10^11^) present in 1 g of stool [17].

The qRT-PCR revealed that we were able to detect between 6.90*10^9^ (RNA Protect) and 8.31*10^9^ (RNA Later) copies of mCherry and between 4.07*10^9^ (RNA Protect) and 4.52*10^9^ (RNA Later) copies of sFGFP per 1 g of stool. Thus per spiked-in *E.coli* cell 7.66 to 9.23 copies of mCherry mRNA and 4.52 to 5.02 copies of sFGFP transcripts were found. So *et al.* [19] showed that induction of the Lac promoter with 1 mM IPTG (as conducted for our spike-in controls) results in a mean of 52 molecules of mRNA per cell encoding a fluorescent reporter protein. Thus the recovery rate of mCherry was between 14.7 % (RNA Protect) and 17.8 % (RNA Later) while for sFGFP a recovery rate between 8.7 % (RNA Protect) and 9.7 % (RNA Later) was found. The good correlation between the recovery rate for mCherry and sFGFP indicates reproducible sample processing. The spiked-in RNA in our experiments was localized in Gram negative intact cells, thus it mimicked the behavior of mRNA in a stool bacterium most accurately. We are not aware of other studies using spiked-in cells for calculation of mRNA recovery.

Application of *in vitro* transcribed RNA as spike-in control for metatranscriptomics was so far only reported twice [20, 21]. Gifford *et al.* [20] used *in vitro* transcribed RNAs spiked into marine bacterioplankton samples prior to RNA isolation and found extremely low recovery rates of 0.00001 % for their spikes. Based on these results they assumed that the sample sequencing depth was in that low range. Satinsky *et al.* [21] found similar recovery rates for RNA spikes in their metatranscriptome analysis of the phytoplankton bloom in the Amazone river plume. However, the stability of pure extracellular mRNA that is added to a sample and then undergoes numerous extraction steps must be significantly different from that of mRNA protected within a bacterial cell. Bursts of nuclease activity from lysing cells in combination with a temperature increase, both occurring during bead beating, may rapidly degrade the spike-in controls. Moreover Gifford *et al.* combined two methods to enrich mRNA and subsequently linearly amplified the mRNA. Thus multiple enzymatic and experimental steps were conducted before sequencing, each accounting for potential loss of the spike.

By contrast, the recovery rates of spikes in our experiments, ranging from 9-18 %, appear to be reasonable given the complexity of the stool sample and the instability of mRNA. However, they also indicate that 80 to 90 % of *E. coli* mRNA was lost during processing. This fraction may be higher for hard-to-lyse stool bacteria.

### mRNA enrichment and sequencing depth

According to the RIN analysis RNA Later and RNA Protect represented the best and the least well suited reagents, respectively, for RNA stabilization in stool samples. Thus we compared the expression profiles of samples stored in these two reagents at room temperature over a period of 144 h using Illumina sequencing of mRNA. Obviously handling demands and shipping cost dramatically increase when samples have to be stored and shipped on ice. Thus we additionally sequenced samples conserved in RNA Later and stored at 4 °C to evaluate whether the stability of the samples would be improved further or if storage at ambient temperature is sufficient to conserve the RNA in this reagent.

Reads mapping to rRNA were removed using the SortMeRNA database. Table 1 shows the sequencing results and the fraction of mRNA in each sample. Between 56 million and 93 million reads were obtained per sample. In the RNA Later time series samples the number of total reads was stable. Interestingly, for samples stored in RNA Protect read counts increased with storage time. The total read counts for the samples stored 24 and 144 h in RNA Protect were higher (82 and 93 million reads) than those for the 0 h sample (72 million reads). In these two samples the percentage of rRNA was much higher (40 % for 24 h and 69 % for 144 h) than in all other samples (2-16 %) analysed, in accordance with the low RIN numbers (3.3 and 5.3). The low efficiency of rRNA removal in these samples is probably caused by the failure of the subtractive hybridisation process used to eliminate rRNA in the RiboZero approach, which depends on the presence of intact 5′and 3′ends that are targeted by the capture oligonucleotide probes. Partially but not completely degraded rRNA will no longer be removed and thus be sequenced. Exonucleases hydrolyse their targets beginning from the ends and account for the degradation of the 5′ and 3′ ends of the transcripts [22]. Interestingly, for the highly degraded RNAs (e.g. 144 h in RP) a prominent degradation smear in the range of 100–200 bp was observed in the Bioanalyzer runs. This partially degraded RNA is sequenced since no size exclusion step is conducted before library preparation and most likely accounts for the high number of read counts in those samples. However, the fraction of mRNA is much lower and thus the sequencing depth is reduced in RNA Protect conserved samples, especially after 24 h and 144 h of storage at room temperature. Low abundant transcripts may be lost in these samples. This clearly demonstrates the better applicability of RNA Later to prevent RNA degradation in stool samples.

**Table 1:** Sequencing statistics

Sample	Total reads x 10^6^	non-rRNA reads x 10^6^	rRNAs reads x 10^6^	% mRNA
RL_4_0h^a^	62.35	53.46	8.90	85.73
RL_4_24h	58.75	53.15	5.61	90.46
RL_4_144h	56.54	49.01	7.53	86.69
RL_RT_0h^b^	75.05	68.82	6.23	91.70
RL_RT_24h	73.05	61.26	11.80	85.85
RL_RT_144h	66.58	56.07	10.51	84.22
RP_RT_0h^c^	72.28	64.21	8.07	88.84
RP_RT_24h	81.60	48.59	33.01	59.55
RP_RT_144h	92.74	28.33	64.40	30.55
K1^d^	94.39	91.65	2.74	97.10
K2	77.01	75.58	1.43	98.15
K3	78.33	76.91	1.42	98.19

Total reads obtained from Illumina HiSeq sequencing were submitted to the SortMeRNA database to identify rRNA sequences within the dataset

^a^RL, RNA Later; 4, 4 °C

^b^RT, room temperature

^c^RP, RNA Protect

^d^Snap frozen control

Previous studies suffered from a low mRNA enrichment efficiency [2, 11], which strongly reduced sequencing depth. The Ribozero Kit for mRNA enrichment was already demonstrated to effectively remove rRNA in stool samples [10]. Consequently we obtained 97-98 % mRNA for the snap frozen control samples. However, the integrity of the RNA has a significant influence on the efficiency of rRNA removal using the subtractive hybridization approach. Here we show that rRNA removal from significantly degraded samples is inefficient.

There was no significant difference between samples conserved in RNA Later that were stored at room temperature with those that were stored at 4 °C with respect to the total number of reads per sample and the efficiency of mRNA enrichment. The fraction of remaining rRNA was between 8 and 16 % and thus significantly higher than for the snap frozen controls (1 – 2 %), but stable for 144 h both at RT and at 4 °C. This finding is important from a practical point of view, since it indicates that samples conserved in RNA Later may be stored and transported at room temperature for up to 6 days.

### Bioinformatics workflow for the analysis of sequencing reads

Additional file 4: Figure S3 shows the workflow for the analysis of the Illumina sequencing reads of the 12 different metatranscriptomes. Fastaq reads were subjected to quality control and clipping. Clipped reads that passed the quality control were analysed using SortMeRNA and reads assigned to rRNA sequences were removed from the analysis. Non-rRNA reads were mapped against the Human Microbiome Project (HMP) database using bwa [13]. Read counts per strain and read counts per cds were calculated for bwa showing that 60-80 % of reads mapped to the cds deposited in the HMP. Additionally, Kraken [23] was used to classify reads using its standard database consisting of available NCBI genomes. Here, 40-60 % of the reads could be assigned taxonomically. Although the bwa alignment assigned more reads than Kraken, the latter has been shown to be highly accurate, though sacrificing sensitivity [23].

### Correlation between qRT-PCR and sequencing results

**Fig. 4:**
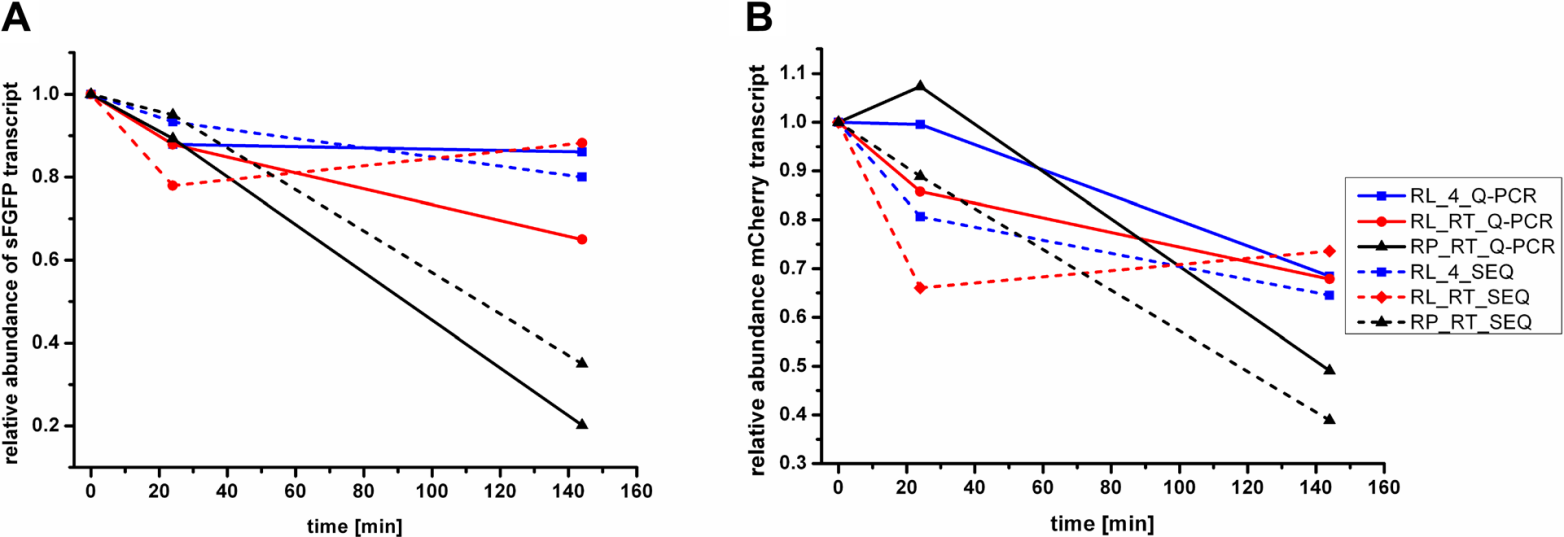
Comparison of transcript abundance determined by qRT-PCR and Illlumina sequencing in mRNA extracted from stool. Samples were conserved in RNA Later or RNA Protect and stored at RT or 4 °C for the indicated time. Relative expression values obtained by RNA sequencing and qRT-PCR are shown for the external spikes sFGFP (**a**) and mCherry (**b**)

The relative abundance of the two spikes mCherry and sFGFP determined by qRT-PCR analysis was compared with their normalized sequencing read counts. Figure 4 shows qRT-PCR and sequencing results for the relative abundance of the two spike-ins mCherry (A) and sFGFP (B) after 0, 24 and 144 h of storage in the stabilizers RNA Protect (RT) and RNA Later (RT, 4 °C). For both spikes the trend of the qRT-PCR analysis was verified by the sequencing results. The relative copy number of the spikes decreased most strongly for RNA Protect, while RNA Later prevented mRNA decay more efficiently. After 144 h of storage sequencing revealed that 38 % (mCherry) and 35 % (sFGFP) of the initial spike was detectable in samples preserved in RNA Protect. This finding correlates well with the results of the qRT-PCR analysis, showing that 20 % (sFGFP) and 49 % (mCherry) of the initial copies of the spike were present after 144 h of storage. For the RNA Later samples stored either at RT or 4 °C, significantly higher proportions of the spikes were still detectable after 144 h storage (80 and 88 % for sfGFP and 65 and 74 % for mCherry) by qRT-PCR. In contrast to the qRT-PCR results, the sequencing analysis showed no significant influence of the storage temperature on mRNA decay of the spike-ins in RNA Later conserved samples. As no size exclusion step is conducted before library preparation for Illumina sequencing, we assume that partially degraded RNAs are still sequenced. This might explain while the qRT-PCR detects differences in mRNA decay between samples stored at ambient temperature and 4 °C in RNA Later that were not found in the sequencing results. Thus, the qRT-PCR approach used here is a more sensitive measure to detect mRNA decay than sequencing. Furthermore this implies that Illumina sequencing of partially degraded mRNA still provides reasonable results for a global profiling of the sample.

### Phylogenetic assignment of sequencing reads using the Kraken software tool

The Kraken program was used to assign phylogenetic labels to the mRNA sequencing reads. Kraken is based on exact alignment of k-mers (a nucleotide sequence of length = k) against a user specified database of genomes and combines high classification accuracy with very fast computational processing [23]. Records consisting of a k-mer and the lowest common ancestor (LCA) of all organisms containing that k-mer in their genomes are the elements of the user-specified database. Sequencing reads were queried against the database and each k-mer present in an individual read is mapped to its lowest common ancestor. The root to leaf node of the taxonomic tree with the highest weight of k-mers mapping to the taxa of this node is used for the classification of the read.

**Fig. 5:**
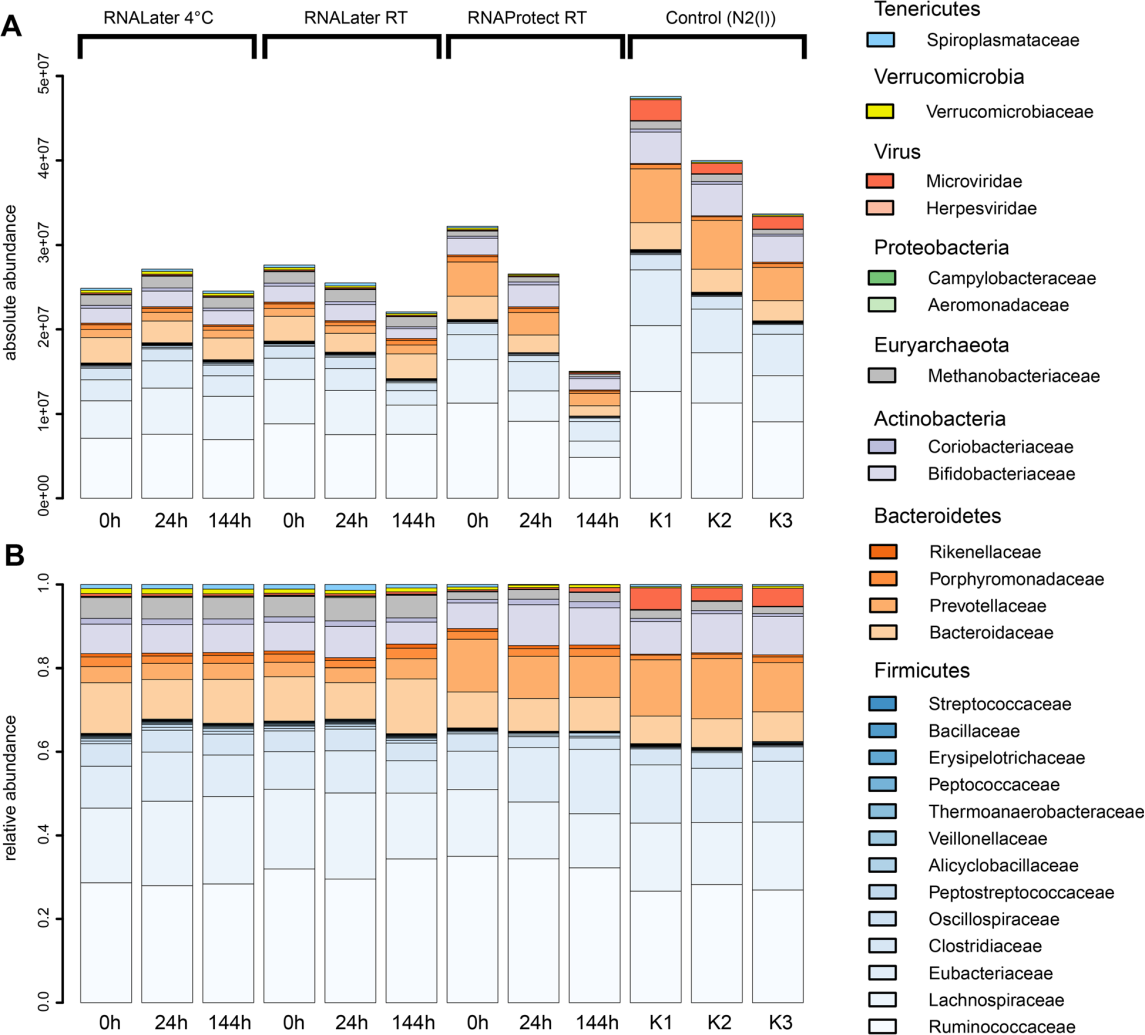
Taxonomic composition of the stool metatranscriptome after storage of the sample in RNA later or RNA Protect for up to 6 days in comparison to snap-frozen controls. Kraken was used to assign taxonomic labels to mRNA sequencing reads on the family level. (**a**) Absolute counts assigned to each family and (**b**) relative abundance of the families within each sample

Here we utilized the k-mer database of viral and bacterial genomes: A total of 98707 genomes representing 5059 different taxa have been downloaded from NCBI Refseq and were used to build the standard Kraken database with k = 31. Non rRNA reads derived from the SortMeRNA filtering were utilized as input for Kraken. They represent both coding and non-coding RNAs. For the sake of brevity the term mRNA will be used subsequently instead of non rRNA. Fig. 5a shows that while the absolute read counts were relatively stable for samples preserved in RNA Later, a strong decrease in taxonomically assignable reads was observed with increased storage time for samples preserved in RNA Protect. Thus, the sequencing depth in RNA Protect decreases during storage as already inferred from the previous analyses. Interestingly, the snap frozen control samples showed a large decrease in total read counts between replicates, from almost 5xe7 for sample 1 to 3xe7 for sample 3. Since those samples were processed in parallel and were not stabilized, the apparent degradation of mRNA may reflect the time spent on ice and highlight the need for speed when isolating mRNA because of the rapid degradation of thawed RNA.

The normalized taxonomic composition of the metatranscriptomes is shown Fig. 5b. The data confirm the stability of RNA Later conserved stool samples, but show that to conserve the taxonomic profile for six days, storage at 4 °C is required. RNA Later and RNA Protect conserved samples showed similar taxonomic profiles in spite of the described differences in RNA stability and mRNA enrichment described above. However, a larger variability was observed in RNA Protect conserved samples during storage as expected. Remarkable shifts occurred between all chemically stabilized samples and the controls K1-K3, which were immediately frozen in liquid nitrogen and represent the gold standard and the original transcriptional profile of the sample.

For example, the relative abundance of Prevotellaceae was enhanced in the controls (approx. 15 vs. 5 %), while the Methanobacteriaceae were more abundant in the RNA Later conserved samples (approx. 7 vs. 3 %). The bias introduced by RNA Protect was smaller, showing the same abundance of Prevotellaceae as the controls. Microviridae, a group of bacteriophages ubiquitously found in fecal samples and waste water [24], comprised a significant fraction of mRNA in the control samples but were absent in chemically stabilized samples. This virus mRNA is apparently lost during nucleic acid isolation from stabilized samples, which is striking since it must be derived from virus replication in the Enterobacteriaceae, which are the virus’s hosts.

### Comparison of Kraken and bwa for taxonomic assignment

Since bwa has been used in most previous metatranscriptome studies, we compared phylogenetic profiles obtained by Kraken (discussed above) with those obtained by bwa (Additional file 5: Figure S4). Both methods identified Firmicutes and Bacteriodetes as the predominant bacterial phyla accounting for the vast majority of transcripts in the stool samples. The sum of the relative abundance of all Firmicutes was approximately 60 % for both taxonomic assignments and the 4 most abundant Firmicutes families were identical for both assignments. However, significant differences between both methods were also observed. bwa identified Clostridiaceae and Eubacteriaceae as the most abundant Firmicutes families, while according to Kraken Ruminococcaceae and Lachnospiraceae represented the predominant Firmicutes families in all samples. In contrast, a higher overall abundance of Bacteriodetes families was observed in the bwa alignment. Remarkably, bwa alignment identified a significant proportion of Fusobacteria which were not found in the Kraken alignment or in the controls. This bacterial phylum has been shown to be associated with colorectal cancer [25] and thus its correct identification is crucial.

Manual inspection of the sequencing reads mapping to the Fusobacteriaceae revealed that most of them had multiple assignments and also mapped to *E. coli*. Since they were only detected in the stabilized samples, but not in the controls which did not receive spike-ins, we conclude that they represented the *E. coli* spike-ins which had been misclassified by BW. Using the default settings of bwa the alignment against the HMP database was performed with 19 k-mers, while Kraken utilizes 31 k-mers. These differences explain the higher accuracy of the Kraken results and are in accordance with the lower sensitivity of Kraken, which taxonomically assigned less reads than bwa. We therefore utilized Kraken for taxonomic classification of reads for all subsequent analyses.

### PCoA and correlation analysis of sample similarity

**Fig. 6:**
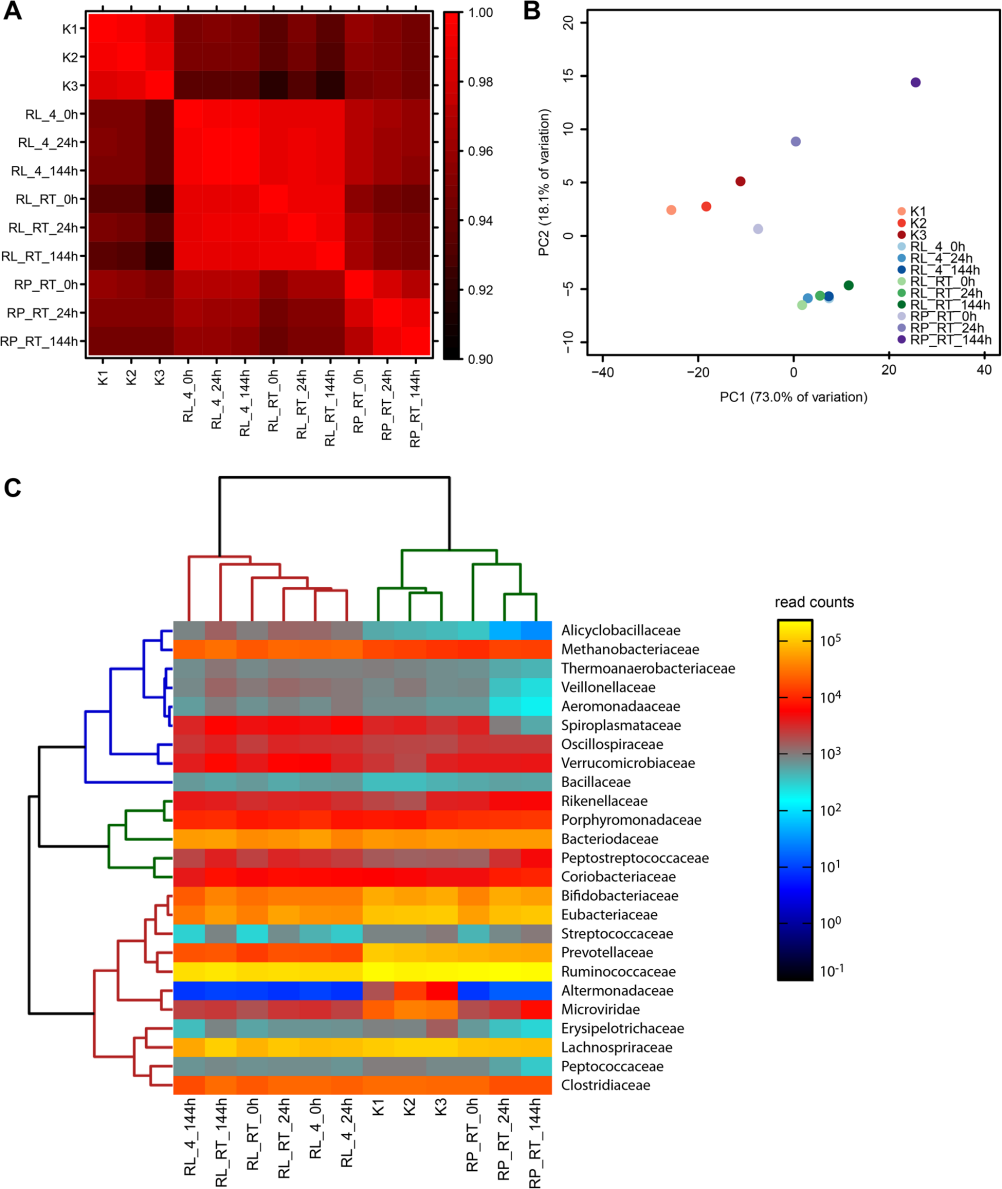
Global comparison of transcriptomes from samples conserved in RNA later or RNA protect. Correlation analysis (**a**), PCoA analysis (**b**) and heat map (**c**) based on the taxonomic classification of the sequencing reads on the family level using Kraken. Hierarchical clustering using Bray-Curtis distance was applied

To identify differences in the transcriptional profiles between the different samples on a global scale and to monitor potential shifts over storage duration, correlation analysis of the taxonomic composition of the microbial communities, as identified with Kraken, was performed using Spearman Correlation. The overall correlation between the samples was high, with a correlation coefficient of 0.92 representing the lowest value between any two samples in the whole data set, indicating a high reproducibility of the experimental approach. Nevertheless, interesting differences between RNA Later and RNA Protect can be observed. Figure 6a shows that transcriptional profiles of all samples stored in RNA Later were highly correlated, regardless of storage duration and temperature. Samples stored in RNA Protect were less well correlated with each other, but more similar to the snap frozen controls. The correlation between the 0 h and 144 h sample was the lowest, indicating substantial changes of the transcriptional profile during storage for 6 days. Moreover, the similarity between the RNA Protect samples and the controls decreased during storage. Thus, RNA Protect conserved the transcriptional profile less efficiently than did RNA Later. Control samples (K1-K3) were highly correlated with each other. Interestingly, samples stored in RNA Protect initially (at 0 h and 24 h) correlated slightly better with the controls than the samples stored in RNA Later.

Figure 6b shows the results of the PCoA analysis using Bray-Curtis similarity. The two axes of the PCoA analysis bundle 73.0 % and 18.1 % of the total variation of the samples, respectively, and most of the variation is thus represented by the first axis. All samples stored in RNA Later built a dense cluster with a relatively low PC1 distance to the controls and no effect of storage duration or temperature, as observed before. For RNA Protect samples, the distance to the controls increased with storage time. The sample taken at t = 0 h showed the highest similarity to the controls of all samples analysed, while the sample stored for 144 h in RNA Protect was the most distant one.

To determine which taxa accounted for the observed differences, a heat map analysis was performed for the normalized read counts assigned to the 25 most abundant bacterial families (Fig. 6c). Bray Curtis Distance was used for a hierarchical clustering of samples and family profiles. Samples could be separated based on the conservation method used: RNA Later, RNA Protect and snap frozen control samples each formed a distinct cluster. RNA Protect samples were closer to the controls than RNA Later conserved samples. Alteromonadaceae and Microviridae transcripts were significantly more abundant in the controls than in the RNA Later or RNA Protect conserved samples. In the RNA Protect conserved samples, reads for Peptococcaceae, Erysipelotrichaceae, Aeromonadaceae, and Veillonellaceae decreased during storage, while reads for Spiroplasmataceae, Peptostreptococcaceae and Microviridae increased. RNA Later samples differed from the controls and the RNA Protect samples with respect to the abundance of Prevotellaceae, Eubacteriaceae, Bifidobacteriaceae and Methananobacteriaceae.

Taken together, these results indicate that RNA Protect is inefficient in conserving the transcriptional profiles over time. Storing the stool sample for 24 h in RNA Protect already altered the transcriptional profile. By contrast, RNA Later is highly efficient in conserving the transcriptional profile of a stool sample for 6 days even at room temperature. However, it introduces a small bias in the transcriptional profile already at t = 0 h, which is maintained throughout storage. Accordingly, RNA Later conserved samples are more dissimilar to the controls than samples stored for less than 24 h in RNA Protect.

### Functional classification of transcriptional profiles

**Fig. 7:**
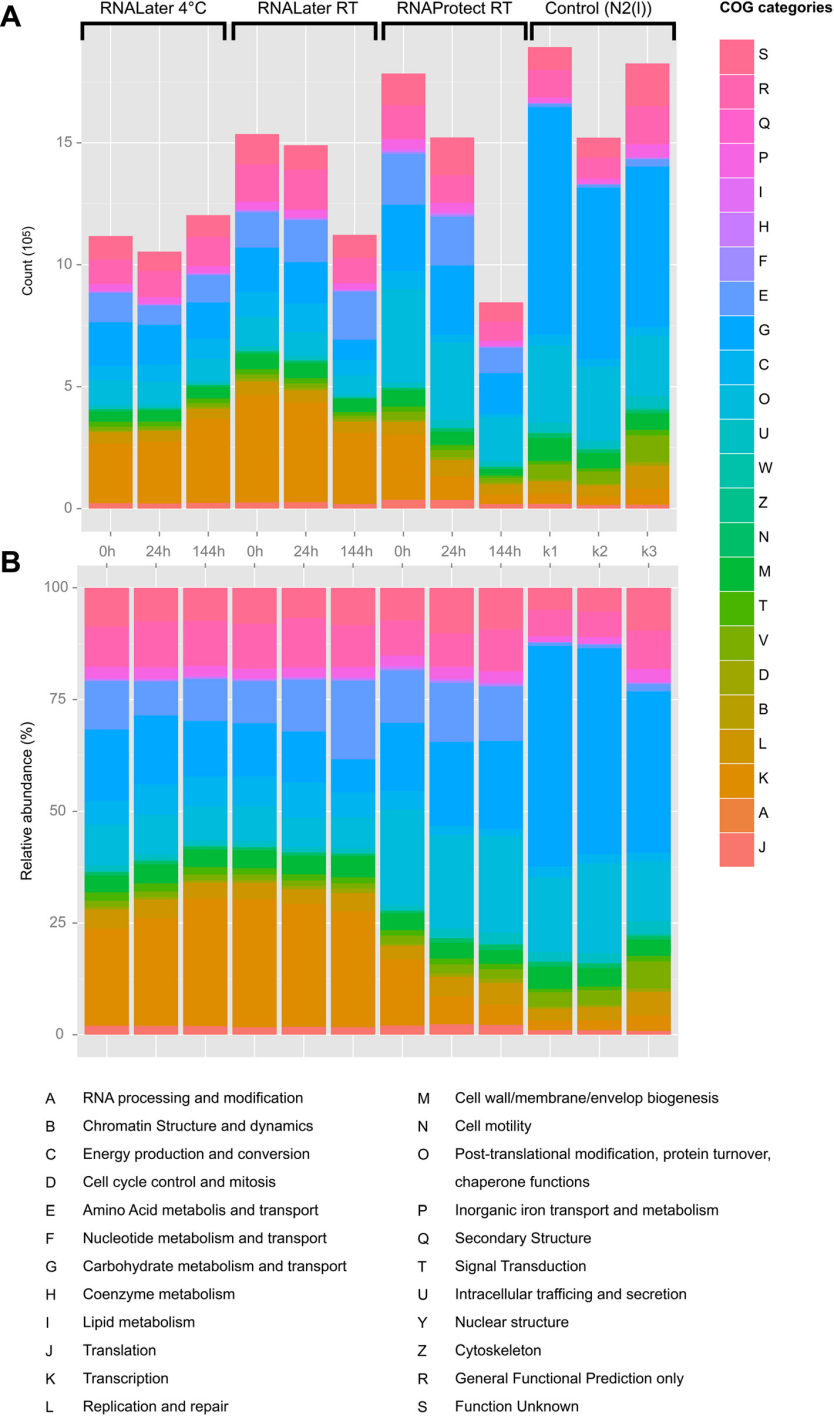
Functional composition of the stool metatranscriptome after storage of the sample in RNA later or RNA Protect for up to 6 days in comparison to snap-frozen controls. COG terms were used for functional classification of sequencing reads. **a** Absolute and (**b**) relative abundance of the reads assigned to the different COG terms

Transcripts mapping to the HMP database using bwa alignment were binned into functional categories according to the COG terms. Absolute and normalized counts assigned to each functional category are shown in Fig. 7 for the 12 different metatranscriptomes. “Transcription”, “Carbohydrate transport and metabolism”, “Amino Acid Transport and metabolism” and “Posttranslational modification, protein turnover and chaperones” represented the categories with the highest number of counts throughout all samples, which is in full accordance with the observed high metabolic activity of the gut microbiota [26, 27]. Again, the functional profiles of samples stored in RNA Later were somewhat different from the control samples while the profiles of samples stored in RNA Protect resembled the profiles of the controls more closely. One very obvious difference between the control samples and all stabilized samples was the higher abundance of COG category “Carbohydrate transport and metabolism” and the reduced abundance of COG category “Transcription” and “Amino acid transport and metabolism” in the controls. Samples stored in RNA Protect showed changes in the abundance of COG categories “Transcription”, “Replication, recombination and repair”,” Cell cycle control, cell division, chromosome partitioning”,” Cell motility” and “Intracellular trafficking” over time, which were not observed in RNA Later conserved samples. The relative abundance of transcripts belonging to the category “transcription” dropped from 14.9 % (0 h) to 4.6 % (144 h) in RNA Protect conserved samples. This shift represents the most pronounced change in the relative abundance of any COG category across the whole data set and highlights the RNA decay in RNA Protect.

The half-life of mRNAs has been shown to be in the range of seconds to minutes [28], differs between species and is influenced by the nutritional status of the cell. Thus, it is absolutely necessary to immediately snap-freeze the stool sample in liquid nitrogen or to immediately resuspend it in a stabilizer solution. Although RNA Later performed best of all tested reagents in preventing mRNA decay, our data indicate that this stabilizer introduces a bias into the transcriptional profile of the sample. As this was observed immediately after resuspension in RNA Later but not in RNA Protect, it cannot be explained by mRNA degradation during sample processing. Within-sample variation is also unlikely to account for this bias, since samples from 3 different parts of the faecium were pooled for one stool sample used for the metatranscriptome analysis. Moreover, all six RNA Later samples were highly similar to each other. The observed differences between the transcriptomes of controls and RNA later conserved samples might be caused by effects of the stabilizing agent on the microbial community during preprocessing of the sample. RNA Later might influence the precipitation behavior of the bacterial cells, thus leading to an enrichment/depletion pattern for some species. Moreover RNA Later contains high amounts of salts introducing osmotic stress to the cells [5]. The lower abundance of Prevotellaceae transcripts in RNA Later preserved samples could have been caused by lysis of some members of this family during sample processing. Prevotellaceae are Gram-negative bacteria and are easier to lyse than the pre-dominant Firmicutes. During the centrifugation step in our protocol, which is used to pellet the bacteria after removal of fecal debris and solid matter, extracellular RNA originating from lysing bacteria may be lost.

For cohort studies with a large number of participants, samples have to be shipped to the labs and cooling of samples is often not feasible. The stabilizer should therefore conserve the transcripts for at least 2–5 days. As we observed a significant decrease in RIN number for RNA Protect conserved samples already after 24 h, this stabilizer is not suitable for such cohort studies. However, if shipping to the laboratory is guaranteed within 24 h and samples are stored on ice, RNA Protect seems to be superior to RNA Later in conserving the original transcriptional profile of the sample.

Recently Franzosa *et al.* [5] addressed the relationship between the oral metagenome, the gut metagenome and the gut metatranscriptome from 8 healthy members of a large cohort study. The samples were self-collected by the donors and stored on ice until delivery to the laboratory within 24 h. Samples were subsequently divided and aliquots were either frozen or fixed in RNA Later or ethanol. The authors mimicked shipping of RNA Later or ethanol fixed samples to the laboratory within 48 h at ambient temperature and compared those meta-omics profiles with the profiles derived from the frozen samples. They found that on the mRNA level the within-subjects correlations between frozen and mock-shipped samples were lower than on the DNA level with respect to species or gene abundance. The lowest value for the Pearson correlation coefficient between treatments on the mRNA level was 0.83. Transcriptional profiles of RNA Later and frozen samples showed a within-subject correlation of approximately 0.93.

In our study, the Pearson correlation coefficient between snap frozen and RNA-Later conserved samples was well above 0.92, yet remarkable differences in the transcriptional profiles were observed. These may not play a large role for a global view on the metatranscriptome of a sample. However, if one is interested in a particular pathogenic species, the activity of a rare microbe, or a specific metabolic pathway, biases such as those occurring in RNA Later or during long-term storage in RNA Protect could be critical. Haiser *et al.* [29] showed that *Eggerthella lenta*, a member of the Actinobacteria, is able to metabolize the cardiac drug digoxin to the inactive form dihydrodigoxin using a specific fumarate reductase. This observation highlights the importance of a single, rare species and a specific pathway for the fate of therapeutics due to the metabolic conversion of host-targeted drugs by the gut microbiota. This example also demonstrates the importance of high sequencing depth to capture transcripts with low abundance.

However a direct comparison between the study of Franzosa *et al.* and our approach is difficult since we utilized a snap frozen control as a reference, while Franzosa *et al*. used frozen samples that were stored on ice for up to 24 h before freezing. Although the authors evaluated the influence of this 24 h storage on ice compared to immediately processed samples based on canine stool, the snap frozen samples represent the more accurate control. Thus, we utilized a more stringent experimental set-up, capable of detecting altered transcriptional profiles between samples and controls more sensitively.

Interestingly, sequencing of highly degraded RNAs originating from samples stored >24 h in RNA Protect still allowed to obtain a transcriptional profile which correlated highly with that of the snap-frozen control sample. Thus, even samples with low RIN numbers can be used for Illumina sequencing. However, due to the reduced fraction of mRNA in those samples, the sequencing depth is reduced and low abundant, but potentially important transcripts might be lost.

## Conclusion

Near-complete RNA extraction and efficient RNA preservation are the basis for successful metatranscriptome analyses that reflect the *in vivo* situation in stool samples. Here we show that among the methods tested the combination of mechanical cell lysis with a bead beater and the utilization of the Powermicrobiome Kit (MoBio) performed best for RNA isolation from stool samples. The established protocol yielded high quality RNA (RIN > 9) suitable for metatranscriptomics. It could easily be adapted to other environmental samples and may be routinely used in large cohort studies.

To evaluate different RNA stabilization methods for stool samples we used *E. coli* spike-in cells expressing fluorescent proteins to mimick mRNA degradation in stool samples during the RNA extraction process. The recovery rates of the spikes were in the range of 9 – 18 % showing the high efficiency of the optimized RNA extraction protocol. With this method, RNA extraction efficiency should be routinely controlled. Quantifying RNA decay either globally (RNA Seq) or for specific targets (Q-PCR, RIN analysis) clearly revealed that RNA Protect is inefficient in stabilizing the transcriptional profile for periods exceeding 24 h. In contrast, the transcriptional profiles of samples preserved in RNA Later did not change significantly even after 6 days storage at ambient temperature. To our knowledge this is the first study providing quantitative data on mRNA and rRNA decay in stool samples. Our data indicate that storage of a stool sample in a preservation reagent (RNA Later) introduces a bias in the mRNA profile. This study represents the first report showing the applicability of Kraken for a complex metatranscriptomic dataset and demonstrates that the Kraken software tool is more reliable than bwa in assigning taxonomic labels to metatranscriptomic data.

The bias introduced by RNA Later on the stool metatranscriptome may vary from person to person depending, among other factors, on the relative abundance of hard-to-lyse Firmicutes versus the relative abundance of Gram negative species that are easier to lyse, e.g. Prevotellaceae or Fusobacteria. The higher stability of the metatranscriptome in RNA Later is obtained at the cost of losing some of the transcripts of labile species. Thus it depends on the focus of the study if this bias is acceptable or not. The take-home message for cohort studies or clinical trials it that RNA Later should be used when stool samples need to be shipped at ambient temperature to the laboratory, since the RNA will be stable for up to 6 days. When shipping can be realized within 24 h or samples can be shipped on ice, RNA Protect may represent a better alternative since the introduced bias is smaller.

### Ethics statement

The study was exempt from full ethics reviews since stool samples were provided by one healthy adult on his/her own free will, no additional information was collected, and there was no risk of revealing person related or medical information. The Ethics Committee of the State Board of Physicians of the German Federal State of Lower Saxony has exempted studies of this nature from full ethics review. Our institution, the Helmholtz Centre for Infection Research, in Braunschweig, Germany, does not require administrative approval of studies exempt from ethics review.

## Methods

### Sampling and pretreatment of fecal samples for RNA extraction

The workflow is shown in Additional file 1: Figure S1. A faecium from a healthy donor was obtained. Approximately 1 g of stool was sampled using a sterile spatula at three different locations of the faecium, suspended in the stabilizer solution, mixed thoroughly, incubated at room temperature for 5 min, and frozen at −80 °C until further processing. This was repeated for each of the four stabilizer solutions and the two storage temperatures (4 °C and RT) to be tested, i.e. a total of 8 stabilized sub-samples were obtained. Snap-frozen controls were immediately frozen in liquid nitrogen. For extraction of RNA, samples were thawed on ice, an equal volume of ice-cold PBS was added and samples were homogenized by vortexing. Samples were centrifuged for 1 min at 4 °C and 700 g to remove solid fecal matter. The supernatant was transferred to a new reaction tube and cells were pelleted at 4 °C and 9.000 g for 5 min. The bacterial cells were resuspended in the different lysis solutions and further treated as described in the special protocols. For comparing the different methods for RNA extraction, a second faecium from the same donor was used and sub-sampled as described above.

### RNA isolation using the MoBio Powersoil Microbiome Total RNA Kit

The ice-cold resuspension of the pelleted cells in the MoBio lysis buffer was added to the supplied bead tubes filled with 500 μl ice-cold phenol-choloroform-isoamylalcohol (PCI) solution (Carl Roth, Germany). Tubes were briefly vortexed to homogenise the mixture. Subsequently the tubes were transferred to a Fast Prep Bead Beater (MP, Germany) and beaten at a speed of 5.5 m/s for 45 s followed by 2 min incubation on ice. The beating procedure was repeated twice. Finally samples were centrifuged for 1 min at 11.000 g at RT and the entire upper aqueous phase was transferred to a new reaction tube (approximately 600–700 μl). Further treatment of samples was according to the manufacturer’s instructions, including a DNAse I treatment on the column. RNA was eluted in 100 μl nuclease free water and the concentration was determined using a Nanodrop ND 1000 (PeqLab, Germany). Integrity of RNA was evaluated using a Bioanalyzer 2100 (Agilent, Germany).

### RNA Isolation using the Norgen Stool RNA Kit

The suspension of bacterial cells in the lysis solution (Norgen, Germany) was either transferred to the supplied bead tubes (unmodified protocol) or to bead tubes containing sterile 0.1 mm zirconia beads (Roth, Germany) (modified protocol); each tube contained 500 μl of ice-cold PCI-solution (see above). Bead beating was performed using the same parameters as described above. After centrifugation (1 min, 11.000 g, RT) the resulting upper aqueous phase was used for RNA extraction according to the Norgen protocol.

### RNA isolation using a lysozyme/mutanolysin pretreatment and the Qiagen RNeasy Kit

Pelleted bacterial cells were resuspended in 200 μl of lysozyme/mutanolysin (LM) solution (1xTE buffer (pH 8.0) containing 15 mg/ml lysozyme (Sigma, Germany) and 500 U/ml mutanolysin (Sigma, Germany)) ([30]). The cell suspension was vigorously shaken for 45 min at 13.000 rpm using an Eppendorf shaker. Afterwards the suspension was transferred to a 15 ml falcon tube containing 50 mg of 0.1 mm zirconia beads (Roth, Germany) and 700 μl RLT lysis buffer (Qiagen, Germany). After vigorous vortexing for 3 min, the beads were removed by centrifugation (11.000 g, 2 min, RT). 470 μl of 100 % ethanol (Roth, Germany) were added and thoroughly mixed with the supernatant which was then applied in two steps to the Qiagen Spin Column. The subsequent RNA extraction procedure was performed according to the manufacturer’s instructions, including the on-column DNase I digestion with the Qiagen DNAse I kit.

### RNA Isolation using the Zoetendal protocol

The protocol of Zoetendal *et al.* [12] was used with a reduced amount of starting material. Instead of 15 g stool, 150 mg of the stool sample were used for RNA isolation, the same amount as applied for the commercial kits. After pretreatment of the stool samples (see above) the Zoetendal protocol was followed. Briefly, the samples were centrifuged (11.000 g, 1 min, RT) after bead beating 3 times (45 s, 5.5 m/s) using a Fast Prep instrument, and the upper aqueous phase was used for phenol/chloroform extraction. The extractions were repeated until the interphase appeared clear. Subsequently an on-column DNAse I digestion using the Qiagen RNEasy Kit was performed. The RNA was washed on the column (according to the RNEasy Mini kit procedure), the column dried (11000 g, 1 min, RT) and the RNA eluted in 60 μl of nuclease free water (Qiagen, Germany). An overnight ethanol precipitation with 1/10 volume 3 M sodium acetate (Life Technologies, Germany), 3 volumes ethanol (Roth, Germany) and 1/100 volume glycogen (Life Technologies, Germany) was carried out. The precipitated RNA was washed 2 times with 70 % ethanol and resuspended in 100 μl of nuclease free water (Qiagen, Germany).

### Cloning of plasmid standards

PCR amplified regions of genes encoding mCherry, Superfolder GFP, glyceraldehyde 3-phosphate dehydrogenase (GAPDH) from *F. prausnitzii* and the 23S rRNA gene of *S. mutans* (for primer sequences see table S1) were cloned blunt-end via the EcoRV restriction site into the pGEM 5Zf(+) vector (Promega, Germany) and the resulting plasmids were transformed in *E. coli* DH5α. Positive clones were selected via blue/white screening on LB agar plates containing 100 μg/ml ampicillin (Sigma, Germany). The clones were cultivated overnight in LB containing 100 μg/ml ampicillin and the plasmids were isolated using the Plasmid Mini Kit (Qiagen, Germany). Cloned plasmids were verified by sequencing.

### Quantitative RT-PCR

For the synthesis of cDNA 1 μg of DNase I treated total RNA was reverse transcribed using the Quantitect Reverse Transcription Kit (Qiagen, Hilden, Germany) in duplicates. Mock reactions replacing the reverse transcriptase with water were used as negative controls to prove the absence of DNA contaminations in the RNA samples and in the components of the kit. The resulting cDNAs were diluted 1:20 and used as templates for PCR. Primers for quantitative PCR were designed using the Primer 3 software (http://frodo.wi.mit.edu). All primers were purchased from MWG Eurofins Operon (Ebersberg, Germany). Table S1 shows the targets and the amplified regions of the primers used in this study. The QuantiTect Sybr Green Kit (Qiagen, Hilden, Germany) was used for quantitative PCR of the cDNA. 15 μl reactions with primer concentrations of 0.25 μM were run in the Light Cycler 480 (Roche, Germany). Threshold (Ct) values were obtained using the Roche software. To determine the primer efficiencies serial dilutions of pooled cDNAs were measured in triplicate for each primer pair. For the determination of the absolute copy numbers of the targets, serial dilutions of cloned plasmid standards with known concentration (determined photometrically using the Nanodrop instrument) were analysed on the same plate as the test samples.

Each sample was measured in triplicate and each experiment was performed at least twice. Data analysis was performed according to Pfaffl *et al.* [31].

### Comparison of different stabilizing reagents

Aliquots of approximately 3.5 g stool originating from the same stool sample and pooled from 3 different locations of the faecium were immediately transferred into 8.5 ml of the different stabilizing agents (RNA Protect, RNA Later, Allprotect, DNA stabilizer). 0.5 ml of an IPTG- induced *E. coli* spike-in, highly expressing mCherry and GFP protein, was added. The total spike-in was calculated to represent less than 1.8 % of the total cell number, assuming that 1 g stool contains approximately 10^11^ cells. The stool samples were immediately and thoroughly resuspended in the stabilizer to avoid the formation of clumps. Pretreatment of stool samples was performed as described above. One part of the samples was then stored at 4 °C, the other at room temperature (see Fig. 2a). Aliquots of 150 mg stool sample were collected in triplicates immediately after resuspension (0 h) and after 2, 6, 12, 24, 144 and 360 h of storage at the two temperatures. After sampling the stool samples were immediately snap-frozen in liquid nitrogen and stored at −80 °C. Total RNA was isolated using the Mobio Kit procedure (see above). mRNA enrichment was carried out with the Ribozero Kit (Epicentre) according to the manufacturer’s instructions, using 4 μg of total RNA solved in 20 μl of nuclease free water (Qiagen, Germany). Enriched mRNA was further analysed using capillary gel electrophoresis (Bioanalyser) to verify removal of 16S rRNA and 23S rRNA.

### Library construction and strand specific RNA sequencing

Paired-end mRNA Seq Illumina libraries were constructed with the Script Seq Illumina Kit. Strand specific paired end sequencing of samples (100 base pairs) was performed on the HiSeq 2000 Sequencer (Illumina, Germany).

### Data analysis using Kraken

For taxonomic classification of the reads we employed Kraken [23] (version 0.10.4-beta) with a k-mer-database of 93560 viral and 5147 bacterial genomes. All genomes, which represent 2718 bacterial and 2343 viral taxa, have been downloaded from NCBI RefSeq on May, 8, 2014 and were used to build the standard Kraken database with k = 31. Reads were assigned in paired end mode with standard parameters. Reports generated from the classifications were used for further custom visualisation. For hierarchical clustering, the abundances were normalized to the number of reads that could be classified. The families Enterobacteriacea and Alcaligenaceae were removed in this analysis since *Escherichia coli* was used as spike-in for the stabilized samples and Alcaligenaceae were found to be mainly represented by reads incorrectly mapping to an Achromobacter genome. The corresponding Achromobacter reference was found to contain an artifact potentially caused by an inappropriate assembly of the reference NC_023061.1. The positions 2434634–2434704 and 6057301–6057337 in this reference were found to be massively covered, whereas more than 99 % of the remaining genome were not covered at all. The origin of these regions is unclear at the moment, but we suspect the reads being an artifact of the Illumina sequencing machines.

### Data analysis using bwa

Filtering of the ribosomal RNA fragments from the sequence output was conducted using SortmeRNA v. 1.8 [32]. The non-ribosomal RNA fragments were mapped against the 382 reference genomes of the HMP gastrointestinal tract database using bwa v. 0.7.5 (−k 19 option for minimum seed length) [13] and SAMtools [33] for storing and filtering nucleotide sequence alignments. For the calculation of hits per CDS and hits per strain of the bwa alignment we employed custom user scripts.

### Statistical analysis

The dataset produced by the Kraken software was used to compare the different samples. Correlation analysis was performed in the R environment applying the Spearman Rank correlation method. Principal Coordinate Analysis (PCoA) was performed with Primer 6 software based on the Bray-Curtis dissimilarities between samples. Hierarchical Clustering of the samples and OTUs was performed with kraken and was also based on the Bray-Curtis-dissimilarity. The heatmap based on this clustering was generated using the hclust script from the MetaPhlAn package.

### Functional analysis using bwa

To obtain functional information about the mRNA reads, transcripts were mapped to the gut specific HMP database using bwa alignment as described above. For genes with known functional annotation clusters of orthologous groups (COG) terms are deposited in the HMP database. Custom user scripts were applied to extract the COG annotation for the mapped reads. COG categorization was then used to bin the mapped mRNA reads into functional categories.

### Availability of Supporting Data

The data sets supporting the results of this article are included within the article and its additional files.

## Additional files

Additional file 1: Figure S1. Workflow for pretreatment of stool samples and RNA isolation using the Fastprep instrument and the Power Microbiome RNA Isolation Kit (MoBio, Germany). Additional file 2: Table S1. Primers used in this study. Additional file 3: Figure S2. Effect of the length of the amplified region on the abundance of transcripts recovered by qRT-PCR after storage of the stool sample in RNA Protect for 6 days at room temperature. Primer pairs amplifying 100, 300, 500 and 700 bp of the spiked-in sFGFP gene, respectively, were used (see Table S1 for primers). Additional file 4: Figure S3. Bioinformatics workflow for analysis of the Illumina sequencing reads. Taxonomic labelling was performed using Kraken and bwa alignment. Functional classifications were assigned to reads according to the COG terms. Additional file 5: Figure S4. Taxonomic classification of sequencing reads using bwa alignment against the Human Microbiome Project database. (A) Absolute counts assigned on the family level; (B) relative abundances of the different families.

## References

[1] Turnbaugh PJ, Ley RE, Hamady M, Fraser-Liggett CM, Knight R, Gordon JI (2007). The human microbiome project. Nature.

[2] Turnbaugh PJ, Quince C, Faith JJ, McHardy AC, Yatsunenko T, Niazi F, Affourtit J, Egholm M, Henrissat B, Knight R, Gordon JI (2010). Organismal, genetic, and transcriptional variation in the deeply sequenced gut microbiomes of identical twins. Proc Natl Acad Sci U S A.

[3] Maurice CF, Haiser HJ, Turnbaugh PJ (2013). Xenobiotics shape the physiology and gene expression of the active human gut microbiome. Cell.

[4] Perez-Cobas AE, Artacho A, Knecht H, Ferrus ML, Friedrichs A, Ott SJ, Moya A, Latorre A, Gosalbes MJ (2013). Differential effects of antibiotic therapy on the structure and function of human gut microbiota. PLoS One.

[5] Franzosa EA, Morgan XC, Segata N, Waldron L, Reyes J, Earl AM, Giannoukos G, Boylan MR, Ciulla D, Gevers D, Izard J, Garrett WS, Chan AT, Huttenhower C (2014). Relating the metatranscriptome and metagenome of the human gut. Proc Natl Acad Sci U S A.

[6] Deutscher MP (2006). Degradation of RNA in bacteria: comparison of mRNA and stable RNA. Nucleic Acids Res.

[7] Shulman LM, Hindiyeh M, Muhsen K, Cohen D, Mendelson E, Sofer D (2012). Evaluation of four different systems for extraction of RNA from stool suspensions using MS-2 coliphage as an exogenous control for RT-PCR inhibition. PLoS One.

[8] Lakay FM, Botha A, Prior BA (2007). Comparative analysis of environmental DNA extraction and purification methods from different humic acid-rich soils. J Appl Microbiol.

[9] He S, Wurtzel O, Singh K, Froula JL, Yilmaz S, Tringe SG, Wang Z, Chen F, Lindquist EA, Sorek R, Hugenholtz P (2010). Validation of two ribosomal RNA removal methods for microbial metatranscriptomics. Nat Methods.

[10] Giannoukos G, Ciulla DM, Huang K, Haas BJ, Izard J, Levin JZ, Livny J, Earl AM, Gevers D, Ward DV, Nusbaum C, Birren BW, Gnirke A (2012). Efficient and robust RNA-seq process for cultured bacteria and complex community transcriptomes. Genome Biol.

[11] Gosalbes MJ, Durban A, Pignatelli M, Abellan JJ, Jimenez-Hernandez N, Perez-Cobas AE, Latorre A, Moya A (2011). Metatranscriptomic approach to analyze the functional human gut microbiota. PLoS One.

[12] Zoetendal EG, Booijink CC, Klaassens ES, Heilig HG, Kleerebezem M, Smidt H, de Vos WM (2006). Isolation of RNA from bacterial samples of the human gastrointestinal tract. Nat Protoc.

[13] Li H, Durbin R (2009). Fast and accurate short read alignment with Burrows-Wheeler transform. Bioinformatics.

[14] Flekna G, Schneeweiss W, Smulders FJ, Wagner M, Hein I (2007). Real-time PCR method with statistical analysis to compare the potential of DNA isolation methods to remove PCR inhibitors from samples for diagnostic PCR. Mol Cell Probes.

[15] Monteiro L, Bonnemaison D, Vekris A, Petry KG, Bonnet J, Vidal R, Cabrita J, Megraud F (1997). Complex polysaccharides as PCR inhibitors in feces: Helicobacter pylori model. J Clin Microbiol.

[16] Mettel C, Kim Y, Shrestha PM, Liesack W (2010). Extraction of mRNA from soil. Appl Environ Microbiol.

[17] Flint HJ, Duncan SH, Scott KP, Louis P (2007). Interactions and competition within the microbial community of the human colon: links between diet and health. Environ Microbiol.

[18] Volkmer B, Heinemann M (2011). Condition-dependent cell volume and concentration of Escherichia coli to facilitate data conversion for systems biology modeling. PLoS One.

[19] So LH, Ghosh A, Zong C, Sepulveda LA, Segev R, Golding I (2011). General properties of transcriptional time series in Escherichia coli. Nat Genet.

[20] Gifford SM, Sharma S, Rinta-Kanto JM, Moran MA (2011). Quantitative analysis of a deeply sequenced marine microbial metatranscriptome. ISME J.

[21] Satinsky BM, Crump BC, Smith CB, Sharma S, Zielinski BL, Doherty M, Meng J, Sun S, Medeiros PM, Paul JH, Coles VJ, Yager PL, Moran MA: Microspatial gene expression patterns in the Amazon River Plume. Proc Natl Acad Sci U S A 2014;111:11085-90. 10.1073/pnas.1402782111 PMC4121788 25024226

[22] Bandyra KJ, Luisi BF (2013). Licensing and due process in the turnover of bacterial RNA. RNA Biol.

[23] Wood DE, Salzberg SL (2014). Kraken: ultrafast metagenomic sequence classification using exact alignments. Genome Biol.

[24] Roux S, Krupovic M, Poulet A, Debroas D, Enault F (2012). Evolution and diversity of the Microviridae viral family through a collection of 81 new complete genomes assembled from virome reads. PLoS One.

[25] Kostic AD, Chun E, Robertson L, Glickman JN, Gallini CA, Michaud M, Clancy TE, Chung DC, Lochhead P, Hold GL, El Omar EM, Brenner D, Fuchs CS, Meyerson M, Garrett WS (2013). Fusobacterium nucleatum potentiates intestinal tumorigenesis and modulates the tumor-immune microenvironment. Cell Host Microbe.

[26] Sekirov I, Russell SL, Antunes LC, Finlay BB (2010). Gut microbiota in health and disease. Physiol Rev.

[27] Mahowald MA, Rey FE, Seedorf H, Turnbaugh PJ, Fulton RS, Wollam A, Shah N, Wang C, Magrini V, Wilson RK, Cantarel BL, Coutinho PM, Henrissat B, Crock LW, Russell A, Verberkmoes NC, Hettich RL, Gordon JI (2009). Characterizing a model human gut microbiota composed of members of its two dominant bacterial phyla. Proc Natl Acad Sci U S A.

[28] Bernstein JA, Khodursky AB, Lin PH, Lin-Chao S, Cohen SN (2002). Global analysis of mRNA decay and abundance in Escherichia coli at single-gene resolution using two-color fluorescent DNA microarrays. Proc Natl Acad Sci U S A.

[29] Haiser HJ, Seim KL, Balskus EP, Turnbaugh PJ (2014). Mechanistic insight into digoxin inactivation by Eggerthella lenta augments our understanding of its pharmacokinetics. Gut Microbes.

[30] Reck M, Rutz K, Kunze B, Tomasch J, Surapaneni SK, Schulz S, Wagner-Dobler I (2011). The biofilm inhibitor carolacton disturbs membrane integrity and cell division of Streptococcus mutans through the serine/threonine protein kinase PknB. J Bacteriol.

[31] Pfaffl MW (2001). A new mathematical model for relative quantification in real-time RT-PCR. Nucleic Acids Res.

[32] Kopylova E, Noe L, Touzet H (2012). SortMeRNA: fast and accurate filtering of ribosomal RNAs in metatranscriptomic data. Bioinformatics.

[33] Li H, Handsaker B, Wysoker A, Fennell T, Ruan J, Homer N, Marth G, Abecasis G, Durbin R (2009). The Sequence Alignment/Map format and SAMtools. Bioinformatics.

