# Supplementary figures and images for: Stool metatranscriptomics: A technical guideline for mRNA stabilisation and isolation
Source: BMC Genomics. 2015 Jul 4;16(1):494. doi: 10.1186/s12864-015-1694-y (PMC4490624; doi:10.1186/s12864-015-1694-y)

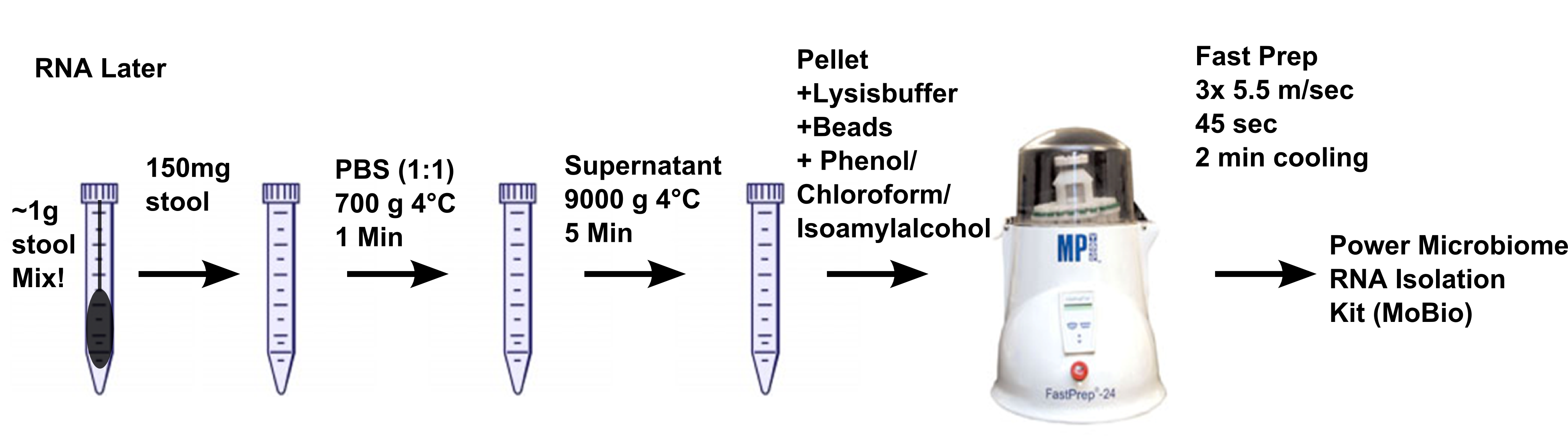

Supplement: Additional file 1: Figure S1. — Workflow for pretreatment of stool samples and RNA isolation using the Fastprep instrument and the Power Microbiome RNA Isolation Kit (MoBio, Germany). [file 12864_2015_1694_MOESM1_ESM.png]

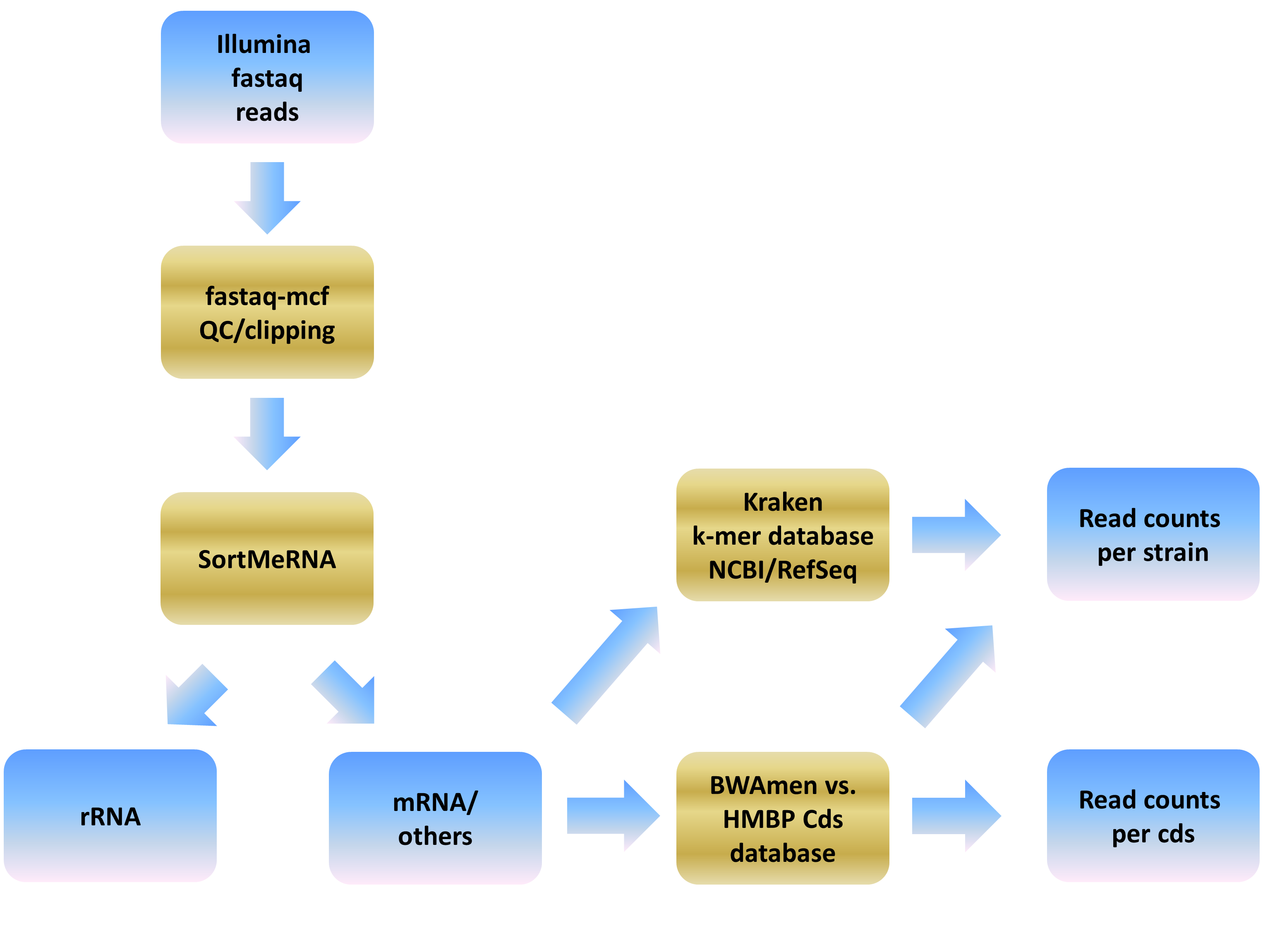

Supplement: Additional file 4: Figure S3. — Bioinformatics workflow for analysis of the Illumina sequencing reads. Taxonomic labelling was performed using Kraken and bwa alignment. Functional classifications were assigned to reads according to the COG terms. [file 12864_2015_1694_MOESM4_ESM.tiff]

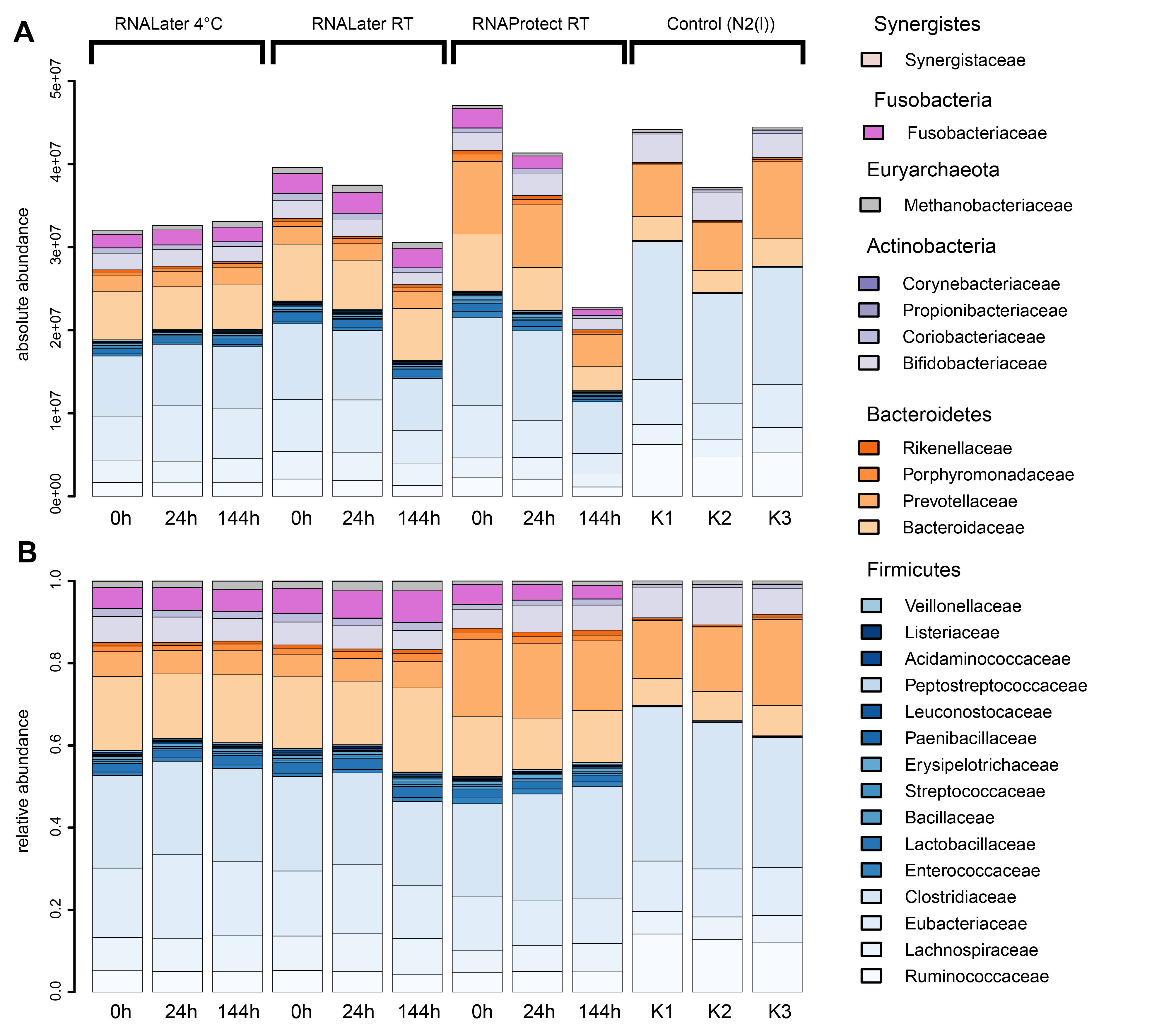

Supplement: Additional file 5: Figure S4. — Taxonomic classification of sequencing reads using bwa alignment against the Human Microbiome Project database. (A) Absolute counts assigned on the family level; (B) relative abundances of the different families. [file 12864_2015_1694_MOESM5_ESM.tiff]
