# Supplementary material for: Stool metatranscriptomics: A technical guideline for mRNA stabilisation and isolation
Source: BMC Genomics. 2015 Jul 4;16(1):494. doi: 10.1186/s12864-015-1694-y (PMC4490624; doi:10.1186/s12864-015-1694-y)
Supplement: Additional file 2: Table S1. — Primers used in this study. [file 12864_2015_1694_MOESM2_ESM.docx]

**Table S1:** **Primers used in this study**

| **Primer** | **Sequence** | **Target gene** | **Product size** |
| --- | --- | --- | --- |
| 23F1075 | gttggcttrgargcagc | 23S rRNA (1075-2241) | 1167 bp |
| 23R2241 | accgccccagthaaact | 23S rRNA (1075-2241) | 1167 bp |
| GAPDH_F | ggatcaggtcatctccgctg | GAPDH *F. prausnitzii* SL 3/3 | 518 bp |
| GAPDH_R | accaagacacgacctgaacc | GAPDH *F. prausnitzii*SL 3/3 | 518 bp |
| MCherry_F | gggcgaggaggataacatgg | mCherry (12-442) | 431 bp |
| MCherry_R | agcccatggtcttcttctgc | mCherry (12-442) | 431 bp |
| sFGFP_F | tgtccgtggagagggtgaa | sFGFP (84-734) | 651 bp |
| sFGFP_R | tggtgatggtgatgggatcc | sFGFP (84-734) | 651 bp |
| sF100_F | tacaagacgcgtgctgaagt | sFGFP (316-418) | 102 bp |
| sF100_R | tgtgtccgagaatgtttcca | sFGFP (316-418) | 102 bp |
| sF300_F | tcacatgaaacggcatgact | sFGFP (228-529) | 302 bp |
| sF300_R | gaacggaaccatcttcaacg | sFGFP (228-529) | 302 bp |
| sF500_F | ggagagggtgaaggtgatgc | sFGFP (91-585) | 495 bp |
| sF500_R | taaaaggacagggccatcgc | sFGFPs (91-585) | 495 bp |
